# Facile Surface Modification of Polyethylene Film via Spray-Assisted Layer-by-Layer Self-Assembly of Graphene Oxide for Oxygen Barrier Properties

**DOI:** 10.1038/s41598-019-39285-0

**Published:** 2019-02-26

**Authors:** Jiwoong Heo, Moonhyun Choi, Jinkee Hong

**Affiliations:** Department of Chemical and Biomolecular Engineering, Yonsei University, 50 Yonsei-ro, Seodaemun-gu, Seoul, 03722 Republic of Korea

## Abstract

The oxygen barrier properties are essential for the food packaging systems that preserve perishable food. In this research, the facile surface modification method for oxygen barrier properties is introduced by using spray assisted layer-by-layer (LbL) self-assembly. The nano-sized graphene oxide (GO−) multilayer films were developed and characterized. Positively charged amine-functionalized GO+ was synthesized using the negatively charged GO− dispersion, ethylenediamine, and 1-(3-dimethylaminopropyl)-3-ethylcarbodiimide methiodide (EDC). Alternating layers of GO− and GO+ were deposited onto the flexible polyethylene (PE) substrate which has no intrinsic gas barrier properties. This method is able to modify surfaces which are challenging for the conventional dipping LbL method. The oxygen transmittance rate of coated PE film (3511.5 cc/m^2^·day) decreased significantly to 1091 cc/m^2^·day after a GO film with a thickness of only 60 nm was deposited. The light transmittance in the visible light range was not significantly decreased after coating of GO films, thus ensuring transparency for PE packaging applications.

## Introduction

Gas barrier properties are essential for films that protect perishable goods such as food or electronics^1–3^. In particular, oxygen barrier properties play a crucial role in food packaging applications, since the most quality loss in foods is due to the oxygen, among the permeable gasses^4^. Polyethylene (PE) has been one of the most extensively applied polymer material because of its advantages in food packaging such as transparency, flexibility, and low cost^5–7^. However, poor gas barrier properties of polymer film limit their application in commercial food packaging field^8^.

Thus, many researchers have devoted significant efforts to enhance the barrier properties of a polymer film by incorporating barrier material forming nanocomposites or by coating barrier film onto the PE surface^9–11^. Nanocomposites consisting of inorganic materials or organoclays demonstrate enhanced oxygen barrier properties, however maintaining their optical clarity is still challenging because of poor dispersion ability of inorganic or organoclay particles^12–15^.

Graphene and graphene oxide (GO) are well-known, atomically thin, two-dimensional barrier materials with excellent gas barrier and mechanical properties^16–18^. The high aspect ratio and planar 2D structure of GO nanoplatelets are known as impermeable to most gasses^19^. Kuila *et al*. research group incorporated GO sheet inside polymer matrix for oxygen barrier film^17^. For the dispersion of GO sheets inside polymer matrix further functionalization process is needed. Depending on the internal structure of the composite film, gas permeance through the film can be increased or decreased. The random orientation of platelets or horizontal in mixed matrix membrane (MMMs) inhibit gas permeation by the tortuous diffusion pathways, while the perpendicular direction of platelets provides faster permeance as they offer fast transportation channels.

Surface coating method with non-permeable materials has been widely applied to oxygen barrier properties. Multilayered films consisting of GO alternatingly deposited with polyelectrolytes such as polyethylenimine (PEI), polyvinyl alcohol (PVA), or polyallylamine hydrochloride (PAH) via layer-by-layer (LbL) self-assembly have been reported^20–22^. LbL self-assembly is a well-developed nanofilm fabrication method that offers precise control over the thickness and internal composition of the films via various complementary interactions, and it can be used with a wide range of materials^23–28^. The multilayer structure of GO can be prepared via LbL self-assembly, and oxygen gas must travel through a tortuous pathway of densely stacked GO layers to permeate the GO coating, thus resulting in a decrease in the OTR^29^.

LbL self-assembly of GO sheets requires complementary materials which can interact with the GO sheets, Chen *et al*. fabricated tens of nanometers-thick GO/polyelectrolyte LbL film onto the 125 μm-thick PET substrates and achieved 99.6% of OTR decrease^25^. Jaime C. research group reported graphene oxide/polymer multilayer film and achieved high oxygen and hydrogen barrier properties^21^. Complementary materials for negatively charged GO should be chosen carefully to make a high performance.

In our work, we designed amine functionalized GO+ for a complementary layer of GO−. Both GO− and GO+ have excellent gas barrier properties, and not only electrostatic interactions between amine groups of GO+ and carboxyl groups of GO− but also pi-pi interaction ensures strong bonding between each layer^18^.

Numerous previous studies on oxygen barrier properties are based on thick polyethylene terephthalate (PET) films, which already have inherent oxygen barrier properties^21^, and there are few reports about modification of thin flexible PE film with nearly no intrinsic oxygen barrier properties. The PE film that we used as a substrate is widely used packaging material with high flexibility and has low oxygen barrier properties itself. Furthermore, conventional dipping LbL self-assembly hardly fabricates stable film onto flexible PE film because of shear stress during dipping process, and oxygen barrier properties were not improved due to the cracks. Therefore, we introduced facile spray-assisted LbL deposition method^30^. Even though the oxygen barrier improvement was comparably not higher than previous researches, we successfully decrease OTR of flexible PE film by coating GO+/GO− multilayer film via spray assisted LbL self-assembly.

Here, we introduced the facile spray-assisted LbL self-assembly of GO without complementary polyelectrolytes on a thin flexible PE substrate. The spray-assisted procedure enables the highly-ordered deposition of a densely-packed multilayer GO film onto a large-area, flexible PE substrate while maintaining high light transmittance.

## Results and Discussion

### Preparation of GO dispersions

**Figure 1:**
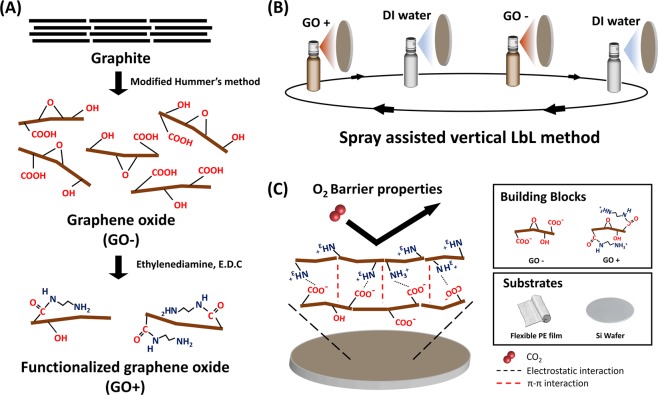
Schematic illustration of the (**A**) preparation method for the two GO dispersions, (**B**) spray-assisted vertical LbL self-assembly method, and (**C**) interactions between the GO+ and GO− layers and available various building blocks and substrates.

GO was prepared by a modified Hummer’s method. GO powder easily dispersed in DI water with the help of ultrasonication to form a negatively charged homogeneous dispersion, because GO has sufficient hydrophilic functional groups, such as hydroxyl, carboxyl, and epoxy groups^31^. To pair with layers of GO−, GO+ was synthesized by covalent attachment of ethylenediamine on GO− (Fig. 1). The amine group of ethylenediamine (NH_2_(CH_2_)_2_NH_2_) reacts with the carboxyl group of GO− to form amide bond via 1-(3-dimethylaminopropyl)-3-ethylcarbodiimide methiodide (EDC) mediated reaction^32^. When amine functionalization, the pH of the GO solution was adjusted to 6.3 to give a proper binding site and to prevent irreversible aggregation.

**Figure 2:**
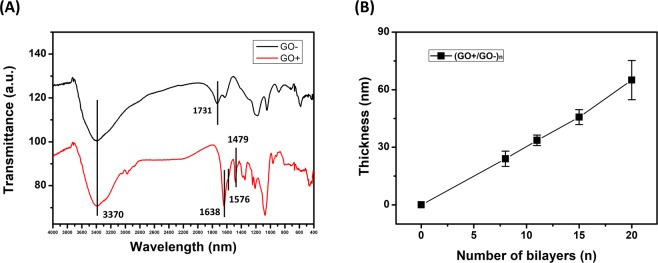
(**A**) FT-IR analysis of GO− (top, black) and GO+ (bottom, red) and (**B**) thickness growth curve of (GO+/GO−)_n_ films on Si wafer.

The functionalization of GO was analyzed by Fourier transform infrared spectroscopy (FT-IR) analysis, as shown in Fig. 2A. The broad OH peak at 3370 cm^−1^ was observed at both of GO− and GO+. The carboxyl group peak at 1731 cm^−1^ shifted to 1638 cm^−1^ because peptide bonds were formed by the reaction between the amine groups of ethylenediamine and the carboxylic groups of GO−. The Amide II band at 1576 cm^−1^ and small sp^3^ C-H peak at 2960 cm^−1^ further proved attachment of ethylenediamine^33^.

### Layer-by-layer self-assembly of GO films

There are three kinds of interactions between GO+ and GO−; electrostatic interactions between amine groups and carboxyl groups, hydrogen bonding that results from hydroxyl groups, epoxide groups and carboxyl groups, and pi-pi stacking interactions. Among these interactions, the electrostatic interaction between GO+ and GO− is a crucial factor for fabricating multilayered GO film with spray assisted LbL self-assembly since the adsorption time is limited in a few seconds. The ionic strengths of GO+ and GO− are governed by pH value. When the pH of GO suspension decreased, the amine groups of GO+ are protonated leading to increased positive charge, while the negative charge of GO− decreased since carboxyl group also protonated. The pH values of freshly prepared GO+ and GO− were 7.8 and 3.4 each and when we tried to fabricate multilayer film at this pH condition, LbL film was not developed. This phenomenon might be caused by insufficient interaction between GO+ and GO−. Therefore, the pH of each solution was adjusted to 6.0 to provide sufficient ionic strength (62.8 mV for GO+ and −69.4 mV for GO−) to build up the multilayer structure.

A schematic illustration of the spray-assisted LbL self-assembly process is shown in Fig. 1B. Initially, the PE substrate is treated by O_2_ plasma, thus activating the PE to contain functional groups such as carboxyl and hydroxyl groups, which result in a negatively charged surface^34^. Then, the PE was fixed onto the glass holder and placed in the vertical direction of spray. GO+ dispersion is sprayed onto the vertically fixed PE substrate, and the GO+ sheets attach to the PE surface due to electrostatic interactions. Weakly bound GO+ impurities are removed thoroughly by spraying with DI water, which ensures strong interactions between the sequential GO+ and GO− layers. Then, the GO− dispersion is deposited onto the GO+ layer by the same method, and multilayered (GO+/GO−)_n_ films are fabricated by repeating this procedure. The robust and compact deposition of alternating (GO+/GO−)_n_ multilayer films can be attributed to not only electrostatic interactions between the GO− and GO+ layers but also π-stacking (Fig. 1C).

### Characterization of GO films

The thickness growth of (GO+/GO−)_n_ films (where ‘n’ represents number of deposited bilayers) on Si wafer is shown in Fig. 2B. The thickness of GO films on Si wafer were measured by using profilometer. Since the stylus tip of profilometer is direct contact with the sample, the reflectance of the surface doesn’t affect the results so that accurate thickness could be obtained. The average thickness of (GO+/GO−)_n_ film on Si wafer was 3.25 nm per bilayer. Since the thickness of a single GO layer is reported to be 0.7–1.3 nm, this result indicates that about 3–4 GO layers are deposited with every bilayer. Furthermore, we analyzed the UV-vis absorbance of the various (GO+/GO−)_n_ films on the PE substrate (Fig. S1). The inset of Fig. S1 shows the effect of the number of bilayers on the absorbance at 223 nm, which increased gradually as the number of bilayers increased. These results confirm that the (GO+/GO−)_n_ multilayered film was successfully deposited onto both Si wafer and PE substrate, even with the short adsorption time during the spray-assisted LbL self-assembly. The transmittance of PE substrate at 650 nm measured by UV-vis was 79.8% and after 15 bilayers GO film coated, transmittance changed to 70.0%. The visible range of light is 390–700 nm and multilayered GO film caused a slight decrease in transparency due to the inherent dark brown color of GO but maintained 70% of light transmission.

**Figure 3:**
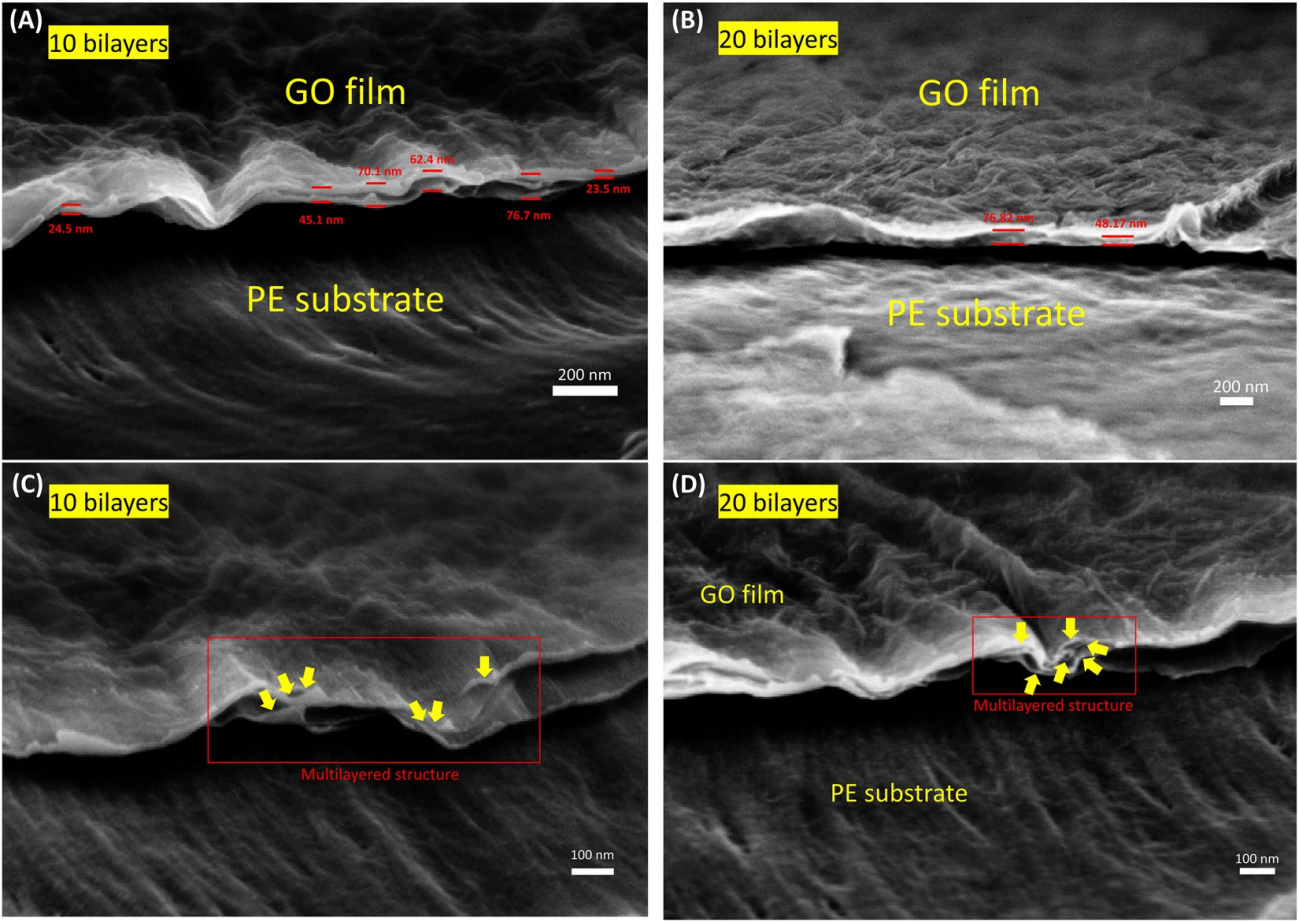
Tilted cross-sectional FE-SEM images of (GO+/GO−)_10_ (**A**,**C**) and (GO+/GO−)_20_. (**B**,**D**).

For the further understanding of GO films, the cross-sectional FE-SEM images were analyzed (Fig. 3). The thickness of (GO+/GO−)_10_ film was about 23.5~76.1 nm (Fig. 3A). External damage might generate the thicker parts such as 70.1 nm and 76.7 nm during sampling. During the sectioning process for cross-section SEM, PE substrates partially wrinkled and multilayered GO sheets peeled off from the stacked state. Thus, the actual thickness of (GO+/GO−)_10_ film seems to approximately 25 nm. In case of the (GO+/GO−)_20_ film, the thickness was revealed about 48.2 nm ~76.8 nm depending on the measured point, and the average thickness of the film was approximately 63.3 nm (Fig. 3B). These results correspond to thickness analysis of GO films on Si wafer (10 bilayers: 32 nm and 20 bilayers: 65 nm). Furthermore, we could see densely packed GO sheets with wrinkled structure. There were no cracks or defects in the GO film, and multilayered GO structure was obviously observed. (Fig. 3C,D)

**Figure 4:**
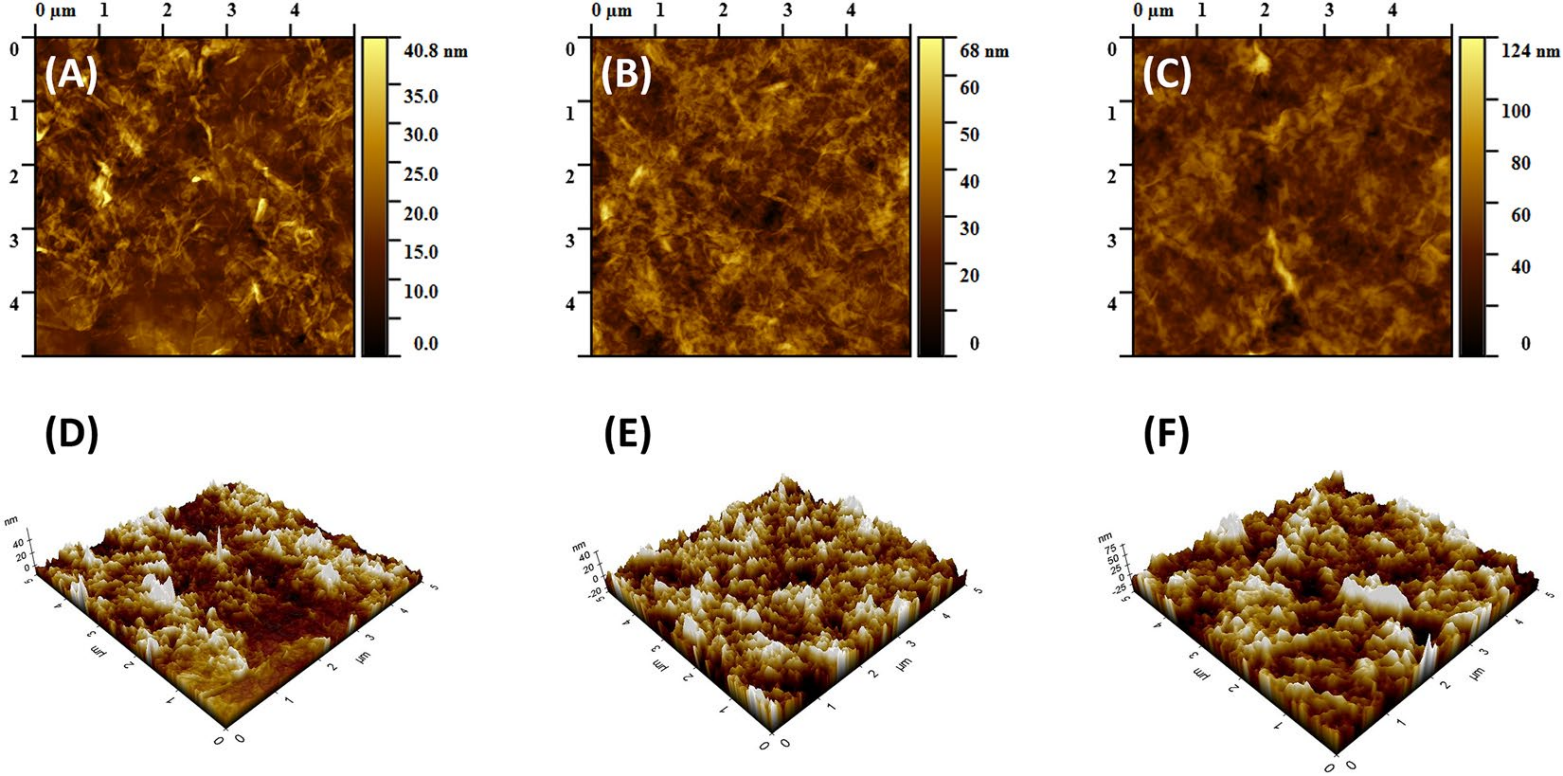
AFM images (**A**–**C**) and 3D AFM images (**D**–**F**) of (GO+/GO−)_n_ films on Si wafer. (**A**,**D**) n = 5, (**B**,**E**) n = 10 and (**C**,**F**) n = 20.

Figure 4 represents the surface morphologies of the (GO+/GO−)_n_ multilayer films. Smooth surfaces and wrinkled GO structures are clearly demonstrated. The roughness of the (GO+/GO−)_n_ films increased with the number of bilayers. The root-mean-square (RMS) roughness of the 5- and 10-bilayer films were 5.83 nm and 7.7 nm, respectively. Even though the 20-bilayer film was rougher than the 5- and 10-bilayer films, its roughness was still only 12.45 nm. These smooth surfaces can be attributed to the sufficient ionic strength of the GO dispersions. The almost fully charged GO sheets prevent aggregation during the spray-assisted LbL process, which enables the compact deposition of GO layers onto the PE film. The alignment of GO sheets in the multilayer film is essential for gas barrier property. By taking advantages of spray-assisted LbL process, we could achieve well-ordered multilayered GO film onto the PE substrate.

### Oxygen barrier properties

**Figure 5:**
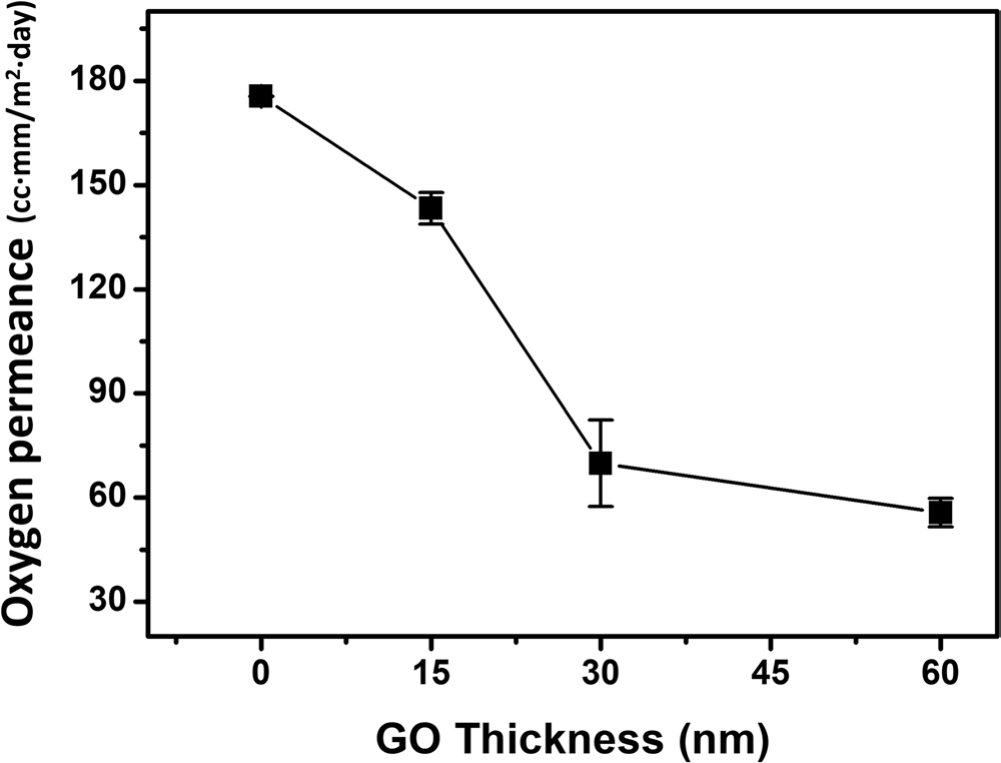
Oxygen permeance of (GO+/GO−)_n_ film.

The oxygen barrier properties of (GO+/GO−)_n_ are shown in Fig. 5 and Table 1. The oxygen barrier performance of both sides of GO coated films was measured, and we found that there was no significant difference in oxygen barrier properties between the upper side and downside of films. This result represents that spray-assisted LbL coating of GO on the PE films not only blocks oxygen permeance from outside but also inside. The GO films which are deposited by spray assisted LbL self-assembly at pH 6.0 condition have densely packed multilayer structure without any cracks or defects (Fig. 3) which are commonly generated other deposition methods such as spin coating, drop-casting, and vacuum filtration. In our system, GO sheets are forming the multilayered structure on the PE film. Different from the single-phase polymer films, our system consists of stacked GO which has a distinct tendency with the polymer film. Until the thickness of GO film reach at 30 nm, as the number of bilayers increased, the surface coverage also gradually increased which represent a drastic increase in oxygen barrier performance. As a result, the bare PE film (50 µm in thickness) showed an OTR of 3511.5 cc/m^2^·day and decreased to 1382.9 cc/m^2^·day after the deposition of the 10-bilayer film. After then, GO was stacked as a multilayer result in a slight increase of oxygen permeance. As the number of bilayers increased from 10 to 20, the OTR further decreased to 1091 cc/m^2^·day. Even though the oxygen barrier improvement was comparably lower than previous researches such as Chen’s work^25^ in which the OTR decreased 99.6% or Jaime’s report^21^ where they achieved superior oxygen and hydrogen barrier performances, we successfully decreased OTR of flexible PE film to 68.94% by coating surface with 60 nm of GO+/GO− multilayer film via spray-assisted LbL self-assembly.

**Table 1:** Oxygen transmission rate of (GO+/GO−)_n_ on PE film. The sample name of ‘up’ means that it is measured from the upper surface of the film and ‘down’ means that it is measure from the other side of the film

Sample	OTR (cc/m^2^·day)	OP (cc·mm/m^2^·day)
Bare PE (up)	3509.67	175.484
Bare PE (down)	3513.33	175.667
(GO+/GO−)_5_ (up)	2801.33	140.067
(GO+/GO−)_5_ (down)	2930.0	146.500
(GO+/GO−)_10_ (up)	1551.7	78.594
(GO+/GO−)_10_ (down)	1214.0	61.045
(GO+/GO−)_20_ (up)	1033.0	52.695
(GO+/GO−)_20_ (down)	1149.0	58.545

.

## Materials and Methods

### Materials

Graphite powder (20 μm), potassium permanganate (KMnO_4_), sulfuric acid (H_2_SO_4_), potassium persulfate (K_2_S_2_O_8_), phosphorus pentoxide (P_2_O_5_), and ethylenediamine were purchased from Sigma-Aldrich. 1-(3-Dimethylaminopropyl)-3-ethylcarbodiimide methiodide (EDC) was purchased from Alfa Aesar. PE film (50 μm in thickness) was obtained from the Yonsei University packaging department.

### Preparation of GO− and GO+

Negatively charged GO (i.e., GO−) was prepared from graphite via a modified Hummer’s method^35,36^. Graphite powder (1 g), K_2_S_2_O_8_ (0.8 g), and P_2_O_5_ (0.8 g) were added to a concentrated H_2_SO_4_ solution (4 mL) with vigorous magnetic stirring. The mixture was stirred for 4.5 h at 80 °C and washed with DI water until the pH of the rinsing water reached 6.0. This pre-oxidized graphite was dried overnight and then added to concentrated H_2_SO_4_ (26 mL) in an ice bath. Then, KMnO_4_ was added slowly to this dispersion, and the temperature was maintained below 30 °C. This mixture was then stirred for 2 h at 36 °C, and DI water (46 mL) was slowly added, followed by stirring for another 2 h at 36 °C. Next, DI water (140 mL) and 30% H_2_O_2_ (2.5 mL) were added to terminate this reaction. The solution was vacuum filtered and rinsed with HCl (400 mL). Finally, residual metal ions and HCl were removed by dialysis for 3 d. GO+ was prepared by the functionalization of GO− suspension. First, the pH of a 100 mL sample of the GO− aqueous dispersion (0.5 mg/mL) was adjusted to 6.3 using NaOH (0.25 M). Excess ethylenediamine (10 mL) was added into this GO dispersion. Then, 1.25 g of EDC was added into the mixture and stirred for 5 h at room temperature. Byproducts and excess ethylenediamine were removed by dialysis for 2 d.

### Spray-assisted layer-by-layer self-assembly

(GO+/GO−)_n_ multilayer films were constructed on a Si wafer (1.5 cm × 3 cm) and PE discs (20 cm diameter) substrates. The Si wafer was thoroughly cleaned using a piranha solution (H_2_SO_4_:H_2_O_2_ = 7:3 v/v) for 5 min, and the PE substrate was cleaned with DI water. Then, the substrates were treated with O_2_ plasma for 2 min. The PE substrate was fixed onto the glass holder and placed in the vertical direction of spray. The GO+ aqueous dispersion (0.5 mg/mL, pH 6.0) was sprayed onto the PE substrate until the solution covered the entire surface. After 20 s, the substrate was sprayed with DI water twice to remove weakly bound GO+ impurities. These steps were repeated with the GO− dispersion (0.5 mg/mL, pH 6.0). The entire procedure was repeated until the desired number of alternating bilayers was obtained, and the samples were labeled (GO+/GO−)_n_, where n represents the number of bilayers. The thickness of the films grown on Si was measured by atomic force microscopy (AFM).

### Characterizations

Dispersion of GO in aqueous solution was achieved via a ultrasonic processor (VC-505, SONICS). The Zeta potentials of GO− and GO+ solution was measured by using SZ-100 Horiba nanoparticle analyzer. The concentration of both GO+ and GO− was 0.5 mg/mL and pH values of GO+ and GO− were adjusted to 6.0 by adding 0.25 M of NaOH or HCl. The thicknesses of the GO films on Si wafer were measured by a profilometer (Dektak 150, Veeco). The functionalization of GO was analyzed by Fourier transform infrared spectroscopy (FT/IR-4700, Jasco). The GO− and GO+ solutions were drop casted onto the Si wafer and dried for the IR measurement. The surface morphologies and roughness of GO films were obtained by non-contact mode atomic force microscopy (AFM, NX-10, Park Systems) and field-emission scanning electron microscopy (FE-SEM, LIBRA 120 microscope, Carl Zeiss). UV-visible absorbance for various numbers of bilayers on PE film was measured from wavelength of 190 nm to 450 nm. The oxygen transmission rate was measured by oxygen permeation analyzer (8001 oxygen permeation analyzer, Illinois Instruments Co.) at standard temperature and pressure condition. The oxygen barrier performance of GO coated PE films was measured on both sides of the PE films.

## Conclusions

In summary, a multilayered GO film was prepared via spray-assisted LbL self-assembly. The spray-assisted procedure enabled the deposition of multilayered GO films onto a large-area, flexible PE substrate which is challenging for the conventional dipping LbL method. GO sheets were deposited on the PE substrates forming densely packed multilayer GO structure. There were no cracks which are the most crucial factor for gas barrier properties. Although the PE substrate had almost no intrinsic oxygen barrier properties, and the thickness of the (GO+/GO−)_20_ film was less than 100 nm, the GO−coated PE film exhibited low oxygen permeability. Our facile spray-assisted LbL self-assembly method paves the way to accelerate the application of nanofilm to an industrial food packaging application.

## Supplementary information

supporting information
