# Supplementary material for: Facile Surface Modification of Polyethylene Film via Spray-Assisted Layer-by-Layer Self-Assembly of Graphene Oxide for Oxygen Barrier Properties
Source: Sci Rep. 2019 Feb 26;9:2754. doi: 10.1038/s41598-019-39285-0 (PMC6391467; doi:10.1038/s41598-019-39285-0)
Supplement: Supplementary file 1 — supporting information [file 41598_2019_39285_MOESM1_ESM.pdf]

Supporting Information Figures

Facile Surface Modification of Polyethylene Film via Spray-Assisted Layer-by-Layer Self-Assembly of Graphene Oxide for Oxygen Barrier Properties

Jiwoong Heo, Moonhyun Choi, and Jinkee Hong*

Department of Chemical and Biomolecular Engineering, Yonsei University, 50 Yonsei-ro, Seodaemun-gu, Seoul 03722, Republic of Korea

*Email address: jinkee.hong@yonsei.ac.kr, Tel.: +82-2-2123-5748

Supporting Information Figure S1

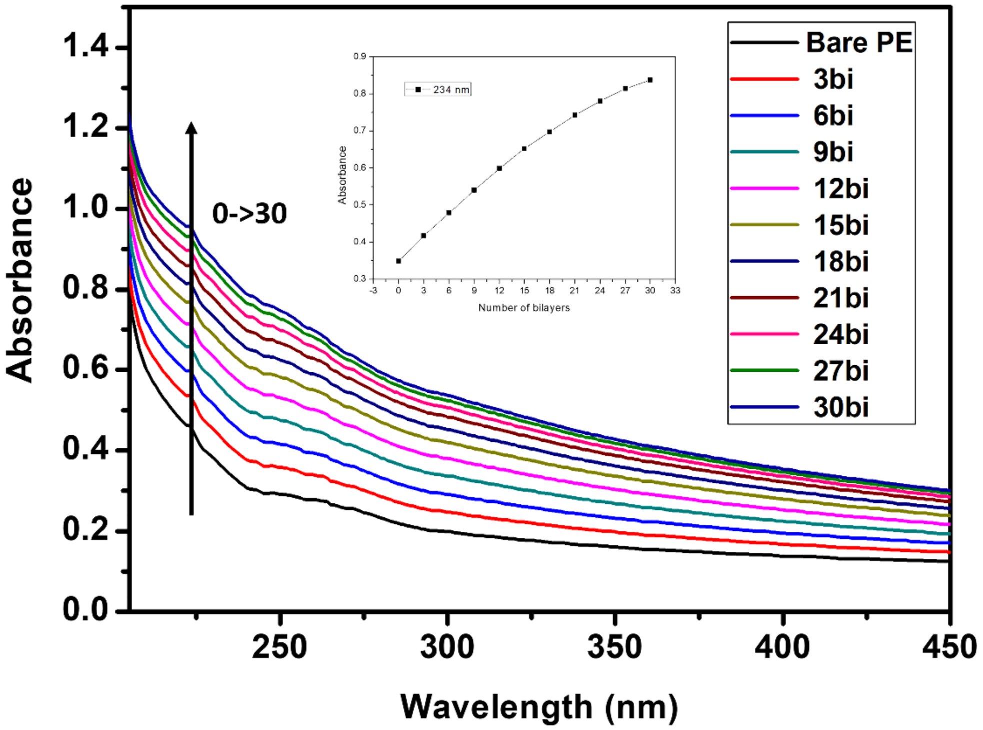

Figure S1. UV-vis absorbance for various numbers of bilayers on PE film. Inset: absorbance at 223 nm as a function of the number of bilayers.

The thickness growth profiles of $(GO+/GO-)_n$ films were measured by UV-vis analysis. The multilayer film gradually deposited onto the PE substrate as the number of bilayers increased. The inset shows the effect of the number of bilayers on the absorbance at 223 nm, which increased gradually as the number of bilayers increased. These results confirm that the $(GO+/GO-)_n$ multilayered film was successfully deposited onto both PE substrate.
